# Supplementary material for: RNA polymerases reshape chromatin and coordinate transcription on individual fibers
Source: bioRxiv. 2023 Dec 23:2023.12.22.573133. Preprint. [Version 1] doi: 10.1101/2023.12.22.573133 (PMC10769320; doi:10.1101/2023.12.22.573133)
Supplement: Supplement 1 [file NIHPP2023.12.22.573133v1-supplement-1.pdf]

bioRxiv preprint doi: https://doi.org/10.1101/2023.12.22.573133; this version posted December 23, 2023. The copyright holder for this preprint (which was not certified by peer review) is the author/funder, who has granted bioRxiv a license to display the preprint in perpetuity. It is made available under aCC-BY 4.0 International license.

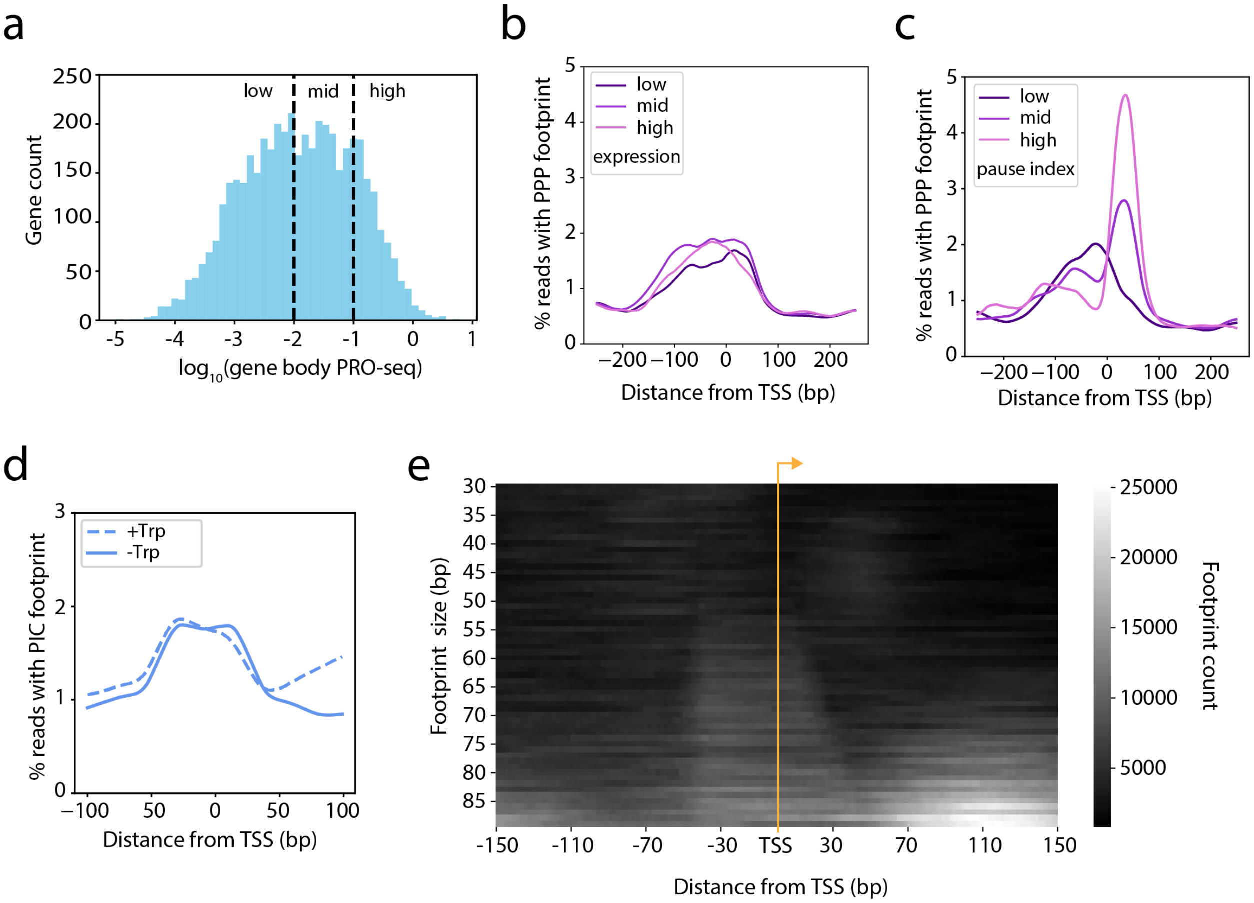

Figure S1 (related to Figure 1)

(a) Histogram showing overall distribution and binning of expression (PRO-seq signal in gene body, normalized by gene length), as used throughout analyses. **(b)** Plot showing enrichment of PPP footprints at genes binned by expression level. **(c)** Plot showing enrichment of PPP footprints at genes binned by pause index**.** **(d)** Plot showing enrichment of PIC footprints with **(solid)** or without **(dashed)** triptolide. **(e)** Heatmap depicting the enrichment of differently sized Fiber-seq footprints with triptolide treatment at positions around transcription start sites (TSS) of genes with a pause index ≥10.

bioRxiv preprint doi: https://doi.org/10.1101/2023.12.22.573133; this version posted December 23, 2023. The copyright holder for this preprint (which was not certified by peer review) is the author/funder, who has granted bioRxiv a license to display the preprint in perpetuity. It is made available under aCC-BY 4.0 International license.

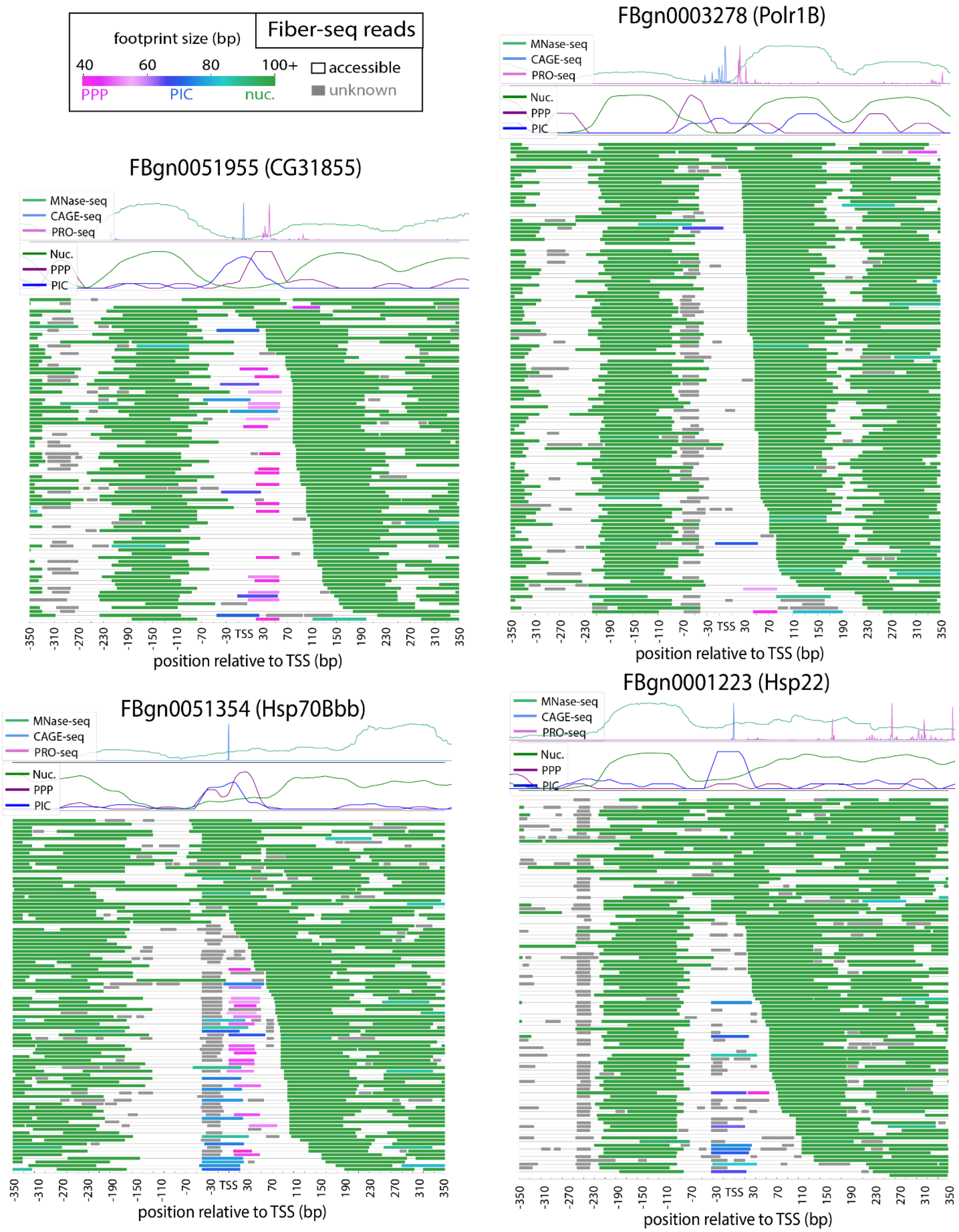

Figure S2 (related to Figure 1)

Fiber-seq reads at four example protein coding loci. Each plot contains a track with MNase-seq, PRO-seq, and CAGE-seq, as well as a track showing enrichment of PPP, PIC, and nucleosome footprints for comparison. Below are all Fiber-seq reads aligned to each locus. Footprints are colored based on predicted identity (PPP = pink, PIC = blue, nucleosome = green, unknown = gray).

bioRxiv preprint doi: https://doi.org/10.1101/2023.12.22.573133; this version posted December 23, 2023. The copyright holder for this preprint (which was not certified by peer review) is the author/funder, who has granted bioRxiv a license to display the preprint in perpetuity. It is made available under aCC-BY 4.0 International license.

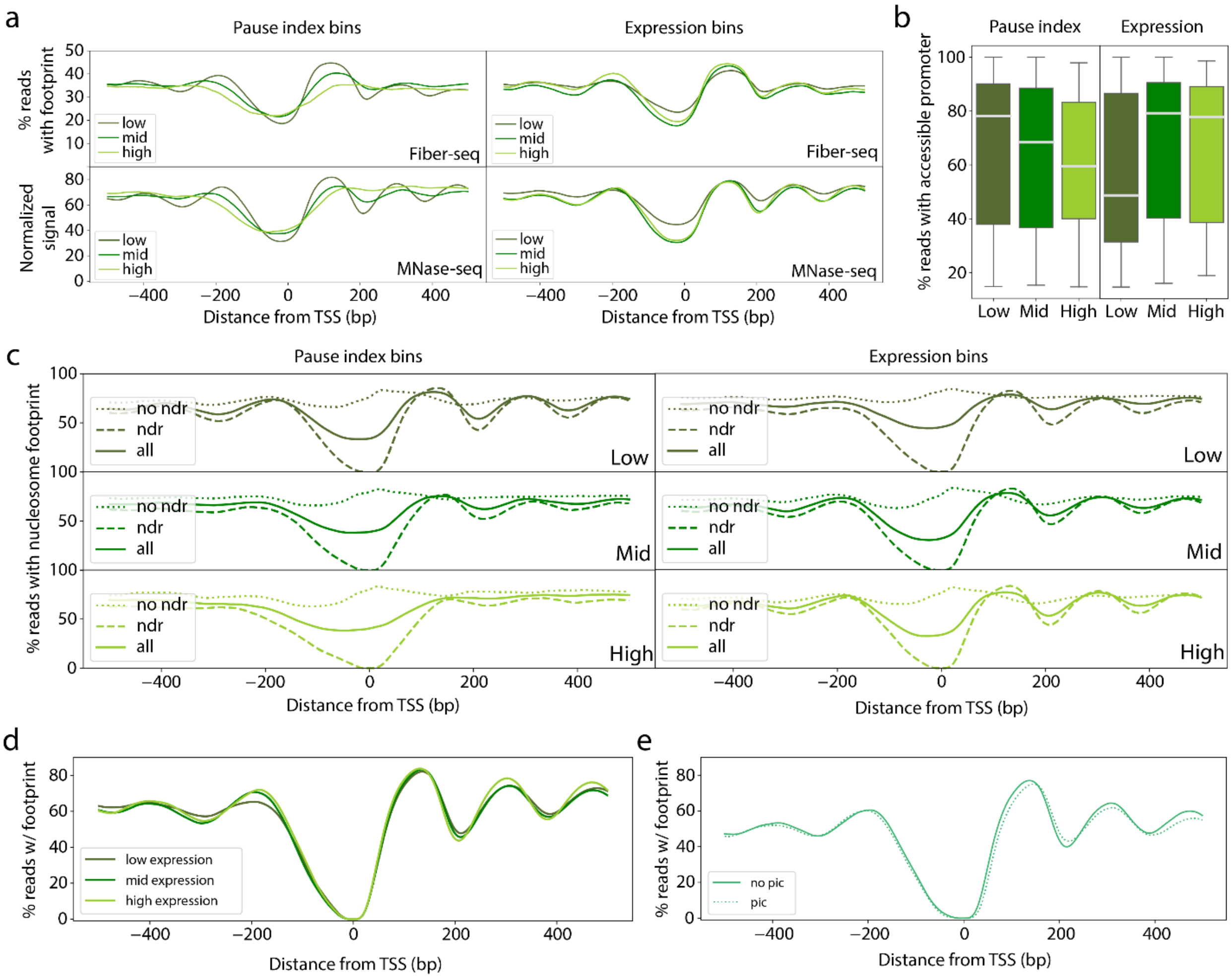

Figure S3 (related to Figure 2)

(a) Comparison between **(top)** MNase-seq signal and **(bottom)** Fiber-seq nucleosome enrichment at **(left)** different pausing levels or **(right)** different expression levels. **(b)** Box plot showing the fraction of Fiber-seq reads with an accessible promoter at **(left)** different pausing levels or **(right)** different expression levels. **(c)** Comparison between Fiber-seq nucleosome enrichment at **(left)** different pausing levels or **(right)** different expression levels with **(dashed)** or without **(dotted)** an accessible promoter, in comparison to the nucleosome enrichment across all genes. **(d)** Enrichment of nucleosome footprints in Fiber-seq reads at different levels of expression, only including reads with an accessible promoter. **(e)** Comparison of nucleosome enrichment in Fiber-seq reads with **(dotted)** or without **(solid)** a PIC footprint, only including reads with an accessible promoter and sampled to capture an equal amount of PIC and no-PIC reads from each gene.

bioRxiv preprint doi: https://doi.org/10.1101/2023.12.22.573133; this version posted December 23, 2023. The copyright holder for this preprint (which was not certified by peer review) is the author/funder, who has granted bioRxiv a license to display the preprint in perpetuity. It is made available under aCC-BY 4.0 International license.

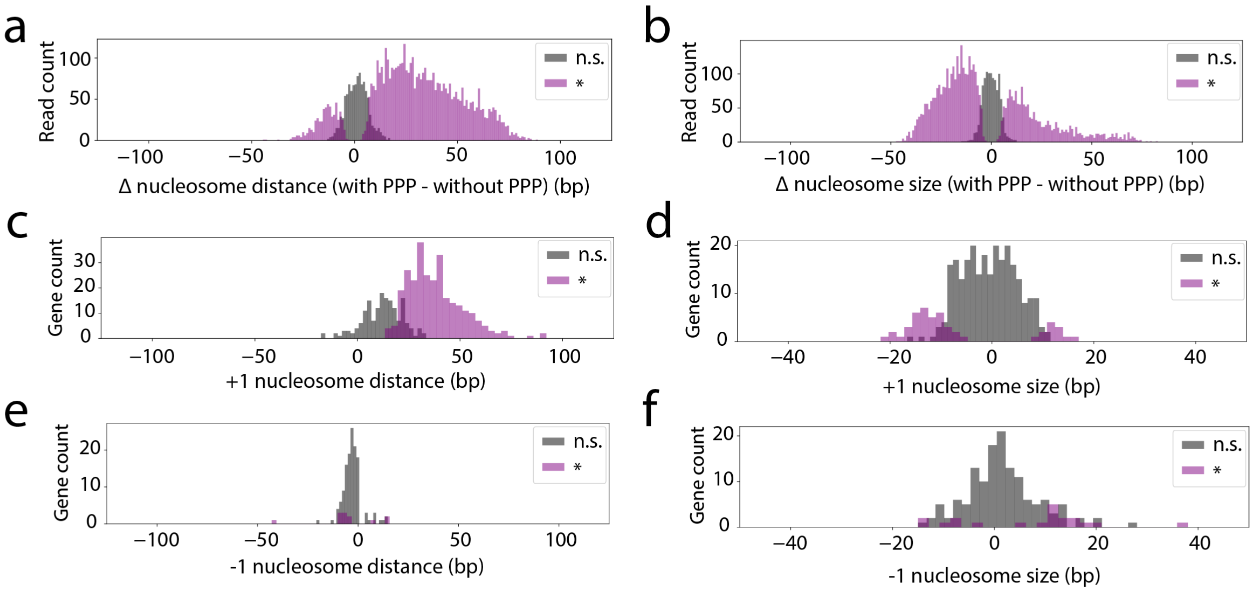

Figure S4 (related to Figure 2)

(a,b) Each individual read with a PPP footprint was compared to the bootstrapped mean distance **(a)** and size **(b)** of the +1 nucleosome. Histogram of difference in +1 distance or size for each PPP read compared to the bootstrapped mean +1 distance or size. Bins are colored and divided based on their associated -log_{10}(p-value), generated by comparing the value to the bootstrapped distribution at the origin locus (empirical confidence interval from bootstrapped mean, n.s. signifies p > .05, * signifies p-value < .05). **(c-f)** Comparing reads with or without a PPP footprint at each locus with at least 5 PPP reads. Graphs in order are **(c)** +1 distance, **(d)** +1 size, **(e)** -1 distance, **(f)** -1 size. Histogram of the mean distance or size of the +1 or -1 nucleosome for reads with a PPP footprint at each locus, shaded based on significance (Wilcoxon Rank Sum test, n.s. signifies p > .1, * signifies p-value < .1).

bioRxiv preprint doi: https://doi.org/10.1101/2023.12.22.573133; this version posted December 23, 2023. The copyright holder for this preprint (which was not certified by peer review) is the author/funder, who has granted bioRxiv a license to display the preprint in perpetuity. It is made available under aCC-BY 4.0 International license.

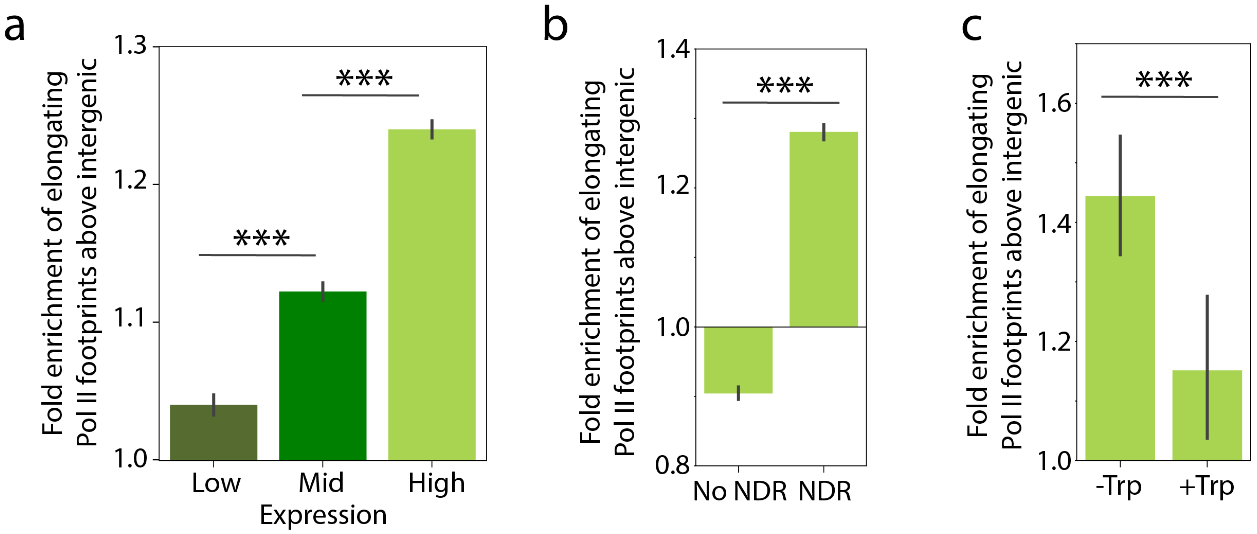

Figure S5 (related to Figure 2)

(a) Bar plot quantifying the fold enrichment of putative elongating Pol II footprints within gene bodies of genes split by expression based on PRO-seq signal within gene bodies Expression values for genes are binned into “high” (≥100), “mid” (≥10, <100), and “low” (<10) values. Pairwise significance is indicated above (two-sample t-test, *** signifies p-value < 0.001). **(b)** Bar plot quantifying the fold enrichment of putative elongating Pol II footprints within gene bodies at reads with an inaccessible or accessible promoter at the corresponding gene. Pairwise significance is indicated above (two-sample t-test, *** signifies p-value < 0.001). **(c)** Bar plot quantifying the fold enrichment of putative elongating Pol II footprints within gene bodies at reads with a PIC footprint at the corresponding gene. Pairwise significance between the -Trp and +Trp datasets is indicated above (two-sample t-test, *** signifies p-value < 0.001).

bioRxiv preprint doi: https://doi.org/10.1101/2023.12.22.573133; this version posted December 23, 2023. The copyright holder for this preprint (which was not certified by peer review) is the author/funder, who has granted bioRxiv a license to display the preprint in perpetuity. It is made available under aCC-BY 4.0 International license.

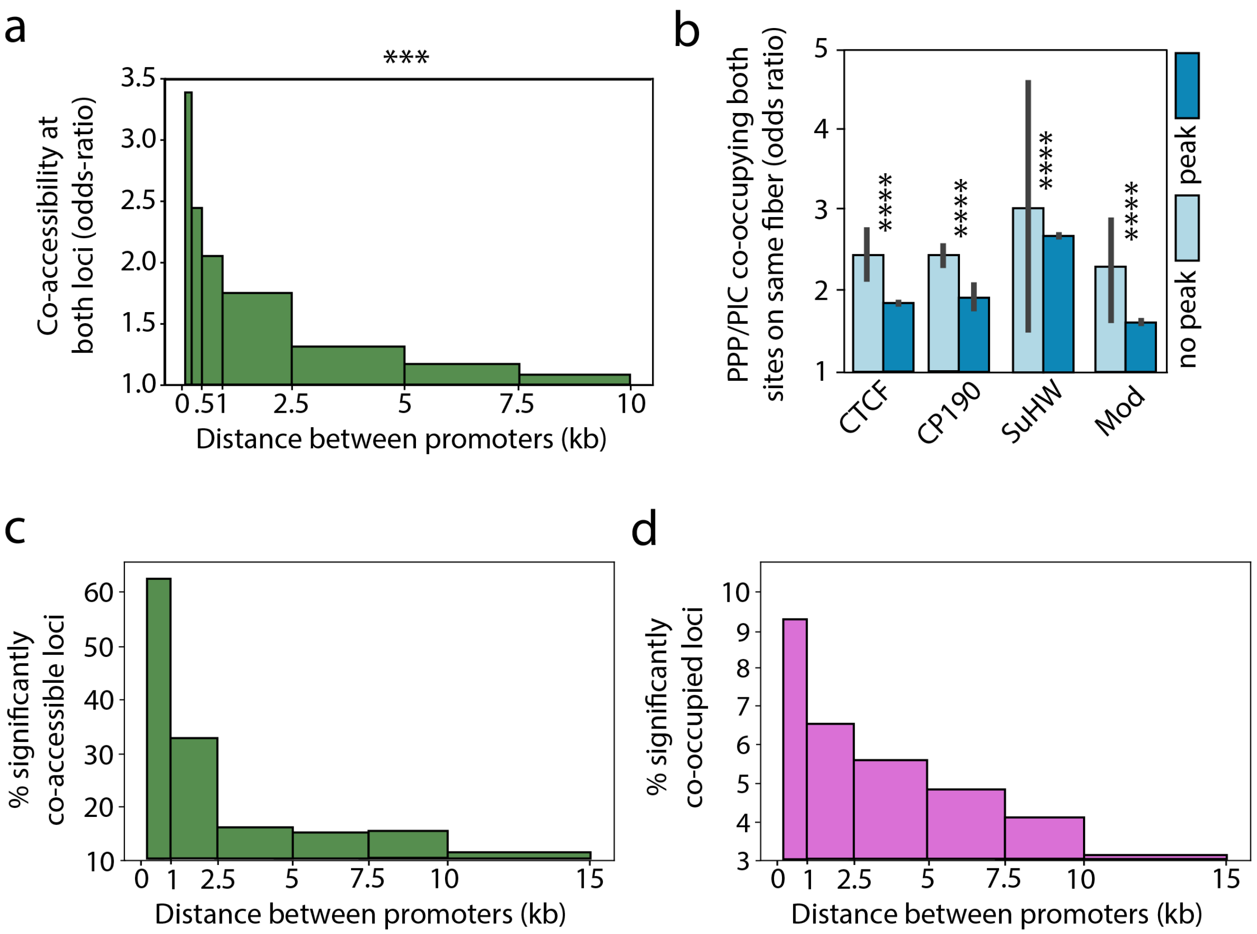

Figure S6 (related to Figure 4)

(a) Bar plot showing the Fisher's exact test odds ratio of co-accessibility of promoters on reads overlapping promoter pairs binned by distance. Significance is schematized above the plot (Fisher's exact test, *** signifies p-value < 0.001). **(b)** Bar plot depicting the coordination of transcription initiation pairs of genes separated or not separated by a ChIP-seq peak of individual insulator binding proteins included in the pooled analysis from Figure 4. All pairs are found within 2.5kb of each other and reads from each pair of groups are sampled to capture an identical count of reads from pairs at any given distance, with error bars corresponding to the confidence interval calculated from 10,000x sampling iterations. Pairwise significance is indicated above (two-sample t-test, **** signifies p-value < 10^{-200}). **(c)** Bar plot showing the percentage of pairs of genes with a significant (p-value < 0.1) Fisher's exact test odds ratio of co-accessibility. **(d)** Bar plot showing the percentage of pairs of genes with a significant (p-value < 0.1) Fisher's exact test odds ratio of co-occupancy by PPP or PIC footprints.

bioRxiv preprint doi: https://doi.org/10.1101/2023.12.22.573133; this version posted December 23, 2023. The copyright holder for this preprint (which was not certified by peer review) is the author/funder, who has granted bioRxiv a license to display the preprint in perpetuity. It is made available under aCC-BY 4.0 International license.

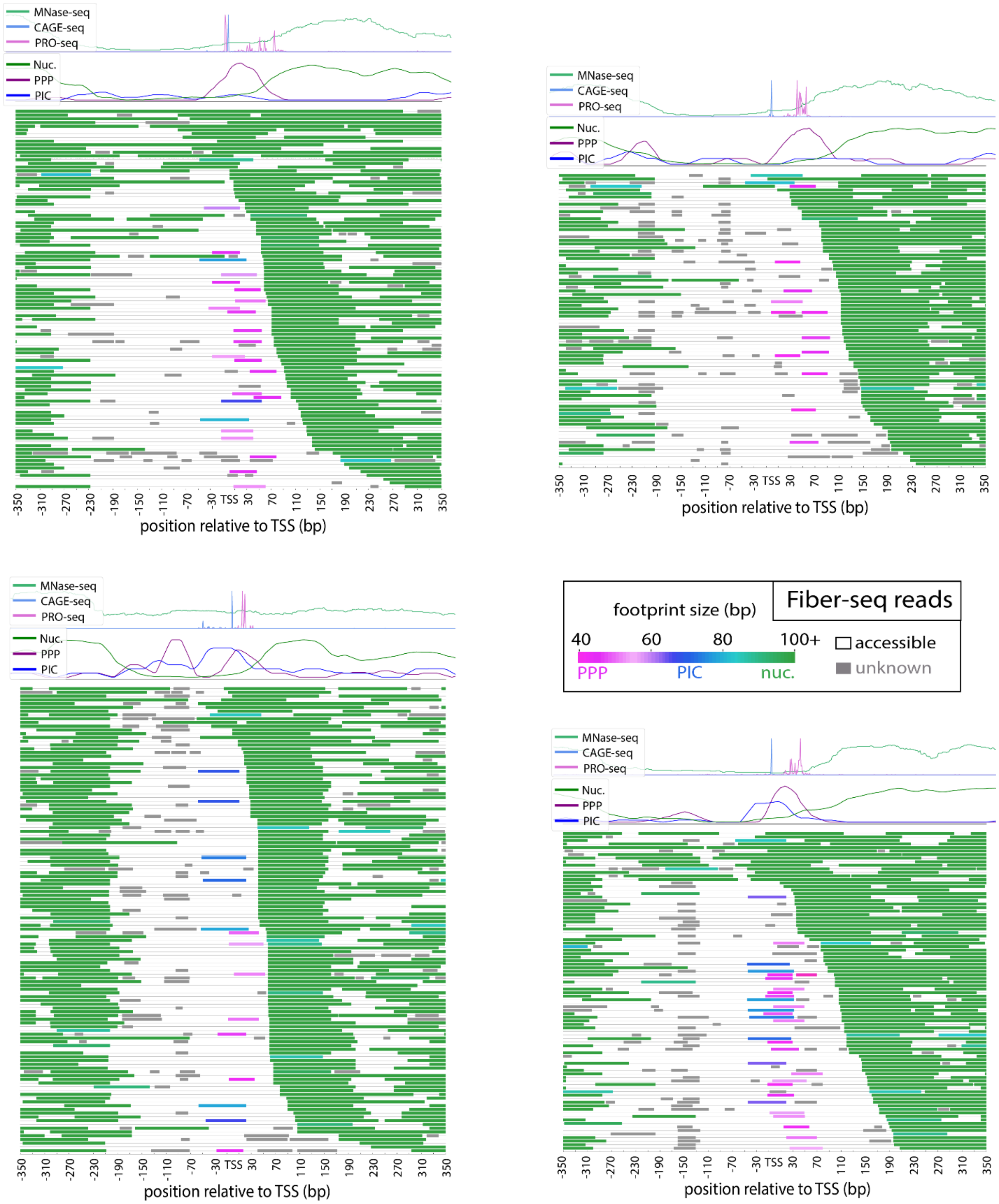

Figure S7 (related to Figure 4)

Fiber-seq reads at four example transcribed enhancer loci. Each plot contains a track with MNase-seq, PRO-seq, and CAGE-seq, as well as a track showing enrichment of PPP, PIC, and nucleosome footprints for comparison. Below are all Fiber-seq reads aligned to each locus. Footprints are colored based on predicted identity (PPP = pink, PIC = blue, nucleosome = green, unknown = gray).

bioRxiv preprint doi: https://doi.org/10.1101/2023.12.22.573133; this version posted December 23, 2023. The copyright holder for this preprint (which was not certified by peer review) is the author/funder, who has granted bioRxiv a license to display the preprint in perpetuity. It is made available under aCC-BY 4.0 International license.

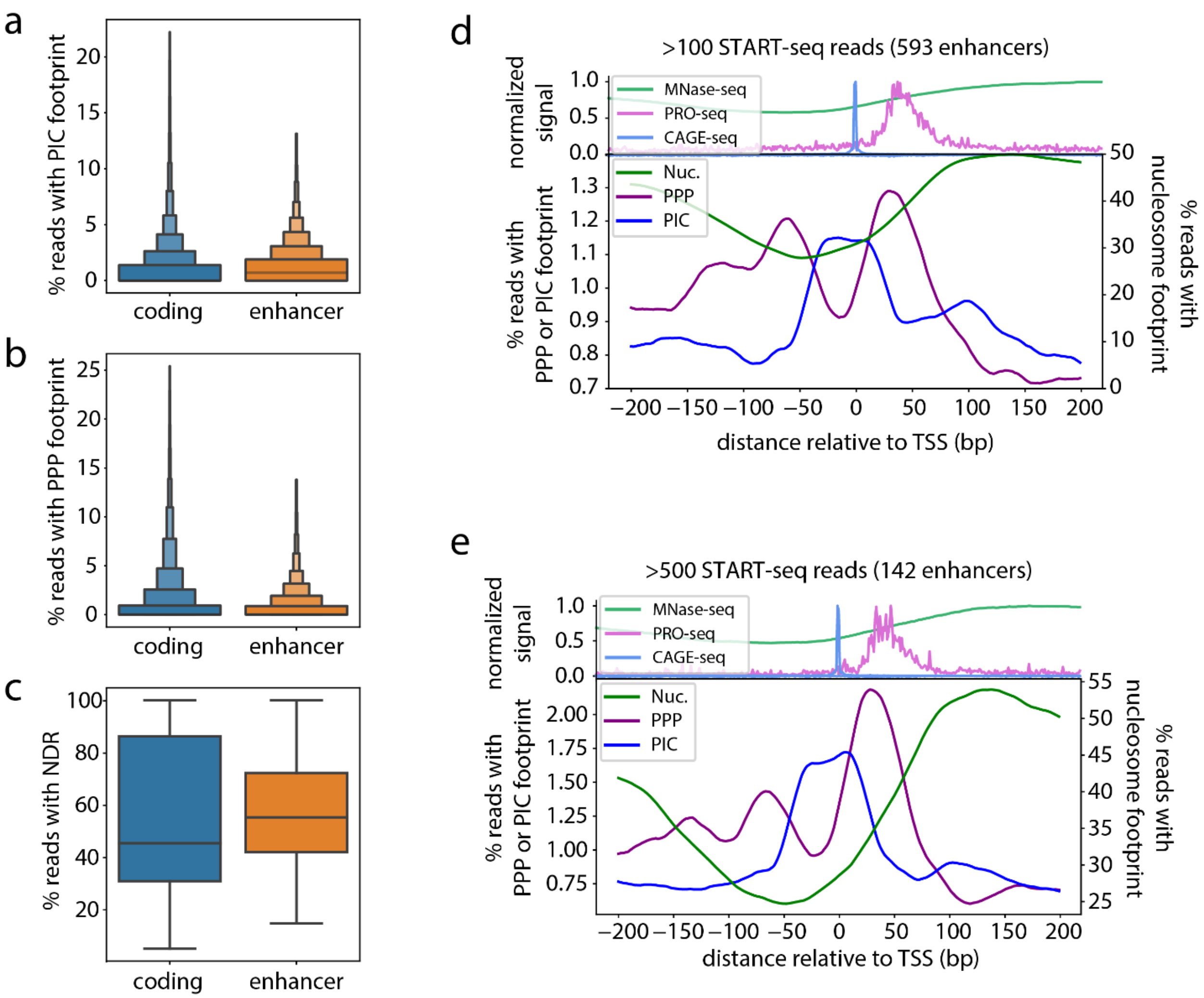

Figure S8 (related to Figure 4)

(a) Boxenplot showing the distribution of percentage of reads with PIC footprints at protein-coding genes and transcribed enhancers. Pairwise significance is indicated above (two-sample t-test, *** signifies p-value < 0.001). **(b)** Boxenplot showing the distribution of percentage of reads with PPP footprints at protein-coding genes and transcribed enhancers. Pairwise significance is indicated above (two-sample t-test, *** signifies p-value < 0.001). **(c)** Boxplot showing the percentage of reads with an accessible promoter for protein coding genes and transcribed enhancers. Pairwise significance is indicated above (two-sample t-test, *** signifies p-value < 0.001). **(d,e)** Plots showing **(top)** MNase-seq, PRO-seq, and START-seq signal at transcribed enhancers compared to **(bottom)** enrichment of PPP, PIC and nucleosome footprints in Fiber-seq reads. Each plot has the indicated (50, 100) minimum coverage of START-seq for the genes included in the plot.

bioRxiv preprint doi: https://doi.org/10.1101/2023.12.22.573133; this version posted December 23, 2023. The copyright holder for this preprint (which was not certified by peer review) is the author/funder, who has granted bioRxiv a license to display the preprint in perpetuity. It is made available under aCC-BY 4.0 International license.

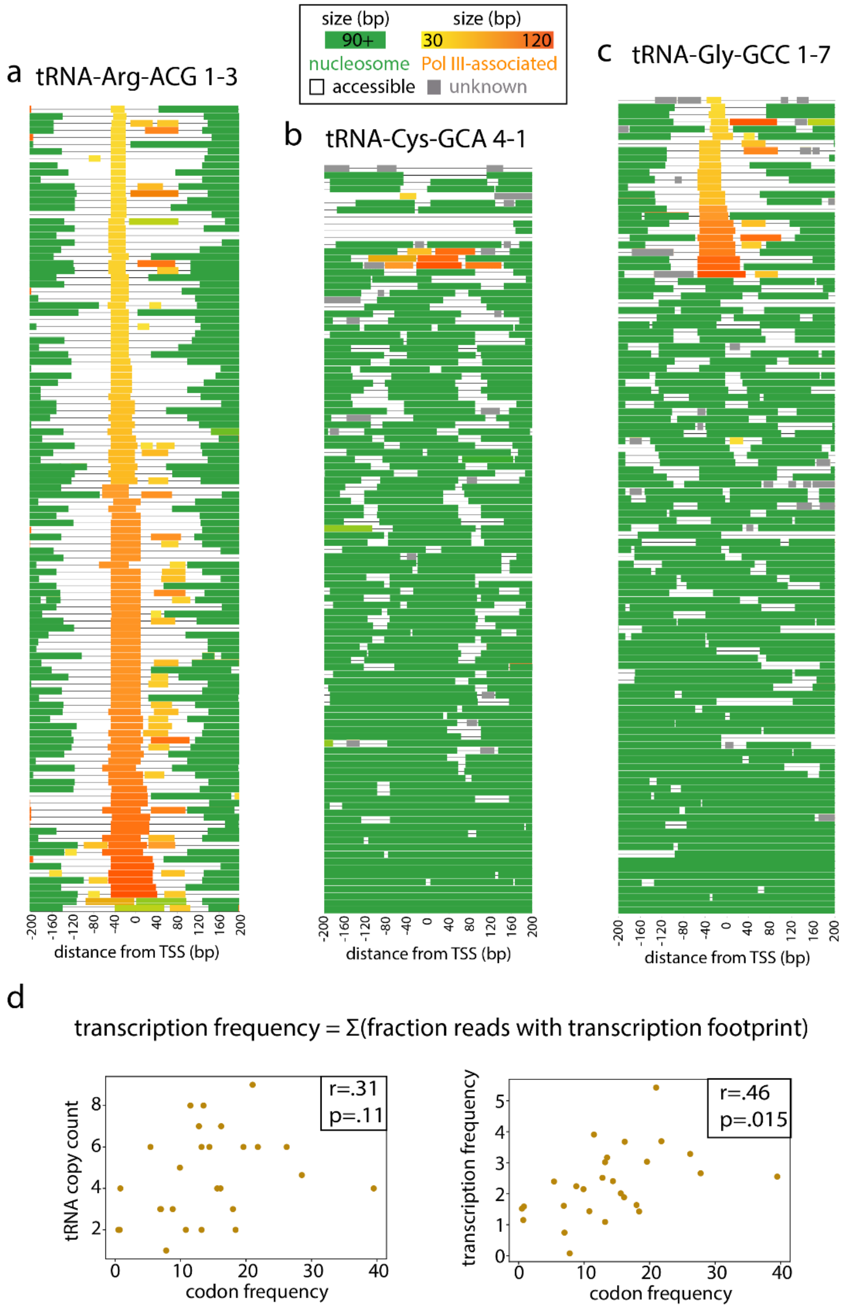

Figure S9 (related to Figure 5)

(a,b,c) Fiber-seq reads at three example tRNA loci. Footprints are colored based on predicted identity (nucleosome = green, tRNA transcription associated footprints = yellow to orange based on size). **(d)** Transcription frequency scores were calculated for each family of tRNAs with identical anticodon sequences and with all members having coverage in the middle 95% of the distribution of overall Fiber-seq sequencing coverage. **(left)** Scatter plot depicting tRNA family gene copy number plotted against their corresponding codon frequency in the *D. melanogaster* genome. **(right)** Scatterplot depicting tRNA isodecoder transcription frequency plotted against corresponding codon frequency in the *D. melanogaster* genome. The Pearson correlation and significance are shown in the top right corner of each plot.

bioRxiv preprint doi: https://doi.org/10.1101/2023.12.22.573133; this version posted December 23, 2023. The copyright holder for this preprint (which was not certified by peer review) is the author/funder, who has granted bioRxiv a license to display the preprint in perpetuity. It is made available under aCC-BY 4.0 International license.

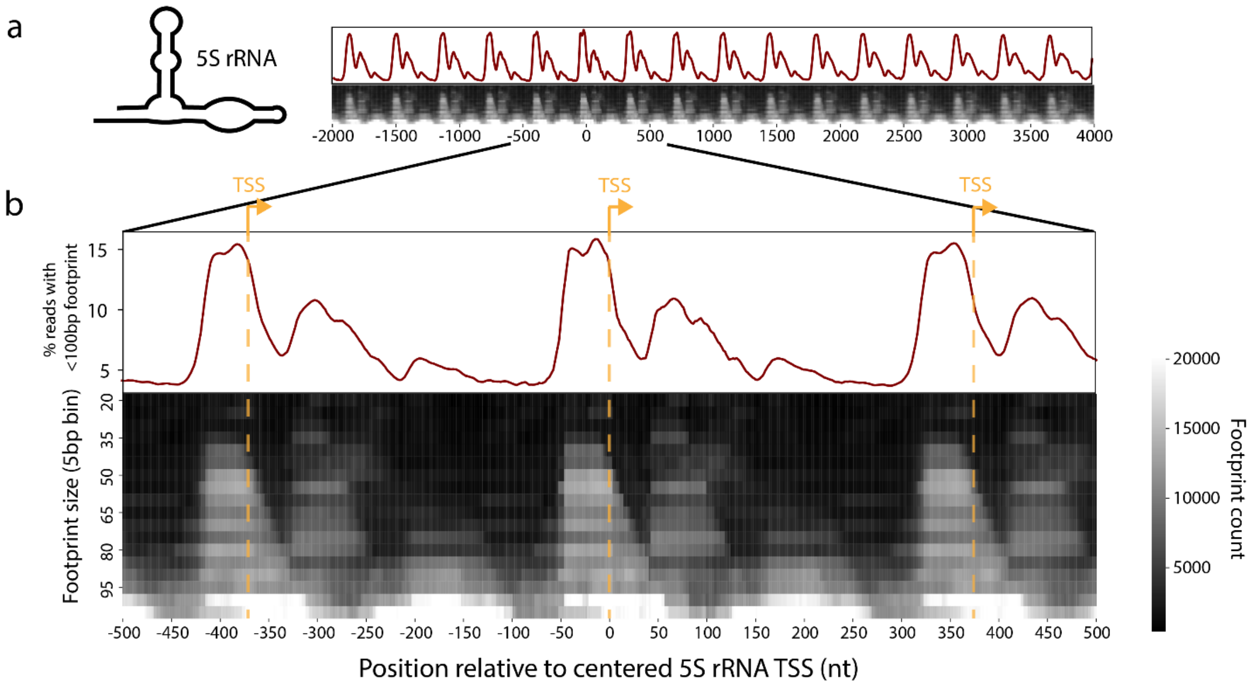

Figure S10 (related to Figure 5)

(a) Heatmaps showing enrichment of differently sized footprints at 5S rRNA genes. **(top)** Wide view showing 16? 5S rRNA copies. **(bottom)** Zoomed in view showing 3 5S rRNA genes. For both wide and zoomed plots: **(top)** enrichment of 30-90 bp footprints relative to 5S rRNA TSSs and (**bottom**) heatmap depicting the enrichment of differently sized Fiber-seq footprints with respect to 5S rRNA TSSs.

bioRxiv preprint doi: https://doi.org/10.1101/2023.12.22.573133; this version posted December 23, 2023. The copyright holder for this preprint (which was not certified by peer review) is the author/funder, who has granted bioRxiv a license to display the preprint in perpetuity. It is made available under aCC-BY 4.0 International license.

Supplemental Tables

Table S1– footprint sizes compared to structures/ChIP

Complex	Organism	Footprint	PDB ID
Elongating Pol II	Yeast	40bp	5C4X
Paused Pol II	Human	45bp	6GML
Elongating Pol II	Human	48bp	6TED
PIC w/ TFIIH	Yeast	70bp	5OQJ
PIC w/ TFIIH	Human	64bp	7NVZ, 7NVY
Core PIC	Human	49bp	7NVS, 7NVT
